# Dubowitz Syndrome Is a Complex Comprised of Multiple, Genetically Distinct and Phenotypically Overlapping Disorders

**DOI:** 10.1371/journal.pone.0098686

**Published:** 2014-06-03

**Authors:** Douglas R. Stewart, Alexander Pemov, Jennifer J. Johnston, Julie C. Sapp, Meredith Yeager, Ji He, Joseph F. Boland, Laurie Burdett, Christina Brown, Richard A. Gatti, Blanche P. Alter, Leslie G. Biesecker, Sharon A. Savage

**Affiliations:** 1 Clinical Genetics Branch, Division of Cancer Epidemiology and Genetics, National Cancer Institute, NIH, Rockville, Maryland, United States of America; 2 Genetic Disease Research Branch, National Human Genome Research Institute, NIH, Bethesda, Maryland, United States of America; 3 Cancer Genomics Research Laboratory, Division of Cancer Epidemiology and Genetics, National Cancer Institute, NIH, Rockville, Maryland, United States of America; 4 Department of Pathology and Laboratory Medicine, David Geffen School of Medicine at UCLA, Los Angeles, California, United States of America; 5 Department of Human Genetics, David Geffen UCLA School of Medicine, Los Angeles, California, United States of America; University of Texas MD Anderson Cancer Center, United States of America

## Abstract

Dubowitz syndrome is a rare disorder characterized by multiple congenital anomalies, cognitive delay, growth failure, an immune defect, and an increased risk of blood dyscrasia and malignancy. There is considerable phenotypic variability, suggesting genetic heterogeneity. We clinically characterized and performed exome sequencing and high-density array SNP genotyping on three individuals with Dubowitz syndrome, including a pair of previously-described siblings (Patients 1 and 2, brother and sister) and an unpublished patient (Patient 3). Given the siblings' history of bone marrow abnormalities, we also evaluated telomere length and performed radiosensitivity assays. In the siblings, exome sequencing identified compound heterozygosity for a known rare nonsense substitution in the nuclear ligase gene *LIG4* (rs104894419, NM_002312.3:c.2440C>T) that predicts p.Arg814X (MAF:0.0002) and an NM_002312.3:c.613delT variant that predicts a p.Ser205Leufs*29 frameshift. The frameshift mutation has not been reported in 1000 Genomes, ESP, or ClinSeq. These *LIG4* mutations were previously reported in the sibling sister; her brother had not been previously tested. Western blotting showed an absence of a ligase IV band in both siblings. In the third patient, array SNP genotyping revealed a *de novo* ∼3.89 Mb interstitial deletion at chromosome 17q24.2 (chr 17:62,068,463–65,963,102, hg18), which spanned the known Carney complex gene *PRKAR1A*. In all three patients, a median lymphocyte telomere length of ≤1^st^ centile was observed and radiosensitivity assays showed increased sensitivity to ionizing radiation. Our work suggests that, in addition to dyskeratosis congenita, *LIG4* and 17q24.2 syndromes also feature shortened telomeres; to confirm this, telomere length testing should be considered in both disorders. Taken together, our work and other reports on Dubowitz syndrome, as currently recognized, suggest that it is not a unitary entity but instead a collection of phenotypically similar disorders. As a clinical entity, Dubowitz syndrome will need continual re-evaluation and re-definition as its constituent phenotypes are determined.

## Introduction

Dubowitz syndrome was originally described in 1965 in a girl with intrauterine growth retardation (IUGR), eczema, short stature, failure to thrive, a high-pitched cry, presumed autosomal recessive inheritance and a distinctive facies (large, low set ears, retrognathia, ptosis, prominent nasal bridge), akin to Bloom and Seckel syndromes [1]. Over time, the phenotype broadened to include varying degrees of developmental and motoric delay, microcephaly, and a variety of minor anomalies [2]. A 1996 review of 141 patients with Dubowitz syndrome by Tsukahara and Opitz defined the syndrome as one with multiple congenital anomalies, cognitive delay, growth failure, an immune defect (allergies and eczema), increased risk of blood dyscrasia (pancytopenia), hematologic malignancy and neuroblastoma [3]. They also speculated that the “extraordinarily broad” phenotypic variability might be due to a metabolic [4] or DNA repair defect. It is important to consider Bloom syndrome, fetal alcohol syndrome, Fanconi anemia and mild Smith-Lemli-Opitz syndrome in the differential diagnosis [5].

In a disorder with unknown pathogenesis, it can be difficult to distinguish a phenocopy from true syndromic heterogeneity. There have been four reports describing specific deletions or mutations in patients with Dubowitz syndrome or a disorder that was etiologically distinct but overlapped with Dubowitz syndrome. The first described a girl with IUGR, tetralogy of Fallot, short stature, microcephaly, cognitive delay, a seizure disorder, facial asymmetry and adducted thumbs. She had a ∼10 Mb deletion at chromosome 13q31.1–13q31.3 and a ∼15 Mb duplication of chromosome 13q31.3–13q33.2 [6]. In a second report, a four-year-old girl with IUGR, microcephaly, poor feeding, ptosis, telecanthus, epicanthal folds, wide nasal bridge, low-set ears, developmental delay, and hoarse high-pitched voice had a ∼2.7 Mb deletion on chromosome 14q32.33. These features also overlapped with the 14q32.3 syndrome, which prompted the authors to speculate that the patient had a phenocopy of Dubowitz syndrome [7]. Martinez diagnosed three children from a consanguineous family with a “Dubowitz-like” syndrome given their IUGR, developmental delay, microcephaly, telecanthus, widely spaced eyes and blepharophimosis. The children did not have a triangular face, hoarse voice, bulbous nose or abnormal ears; eczema was present in one of the children. A homozygous splice mutation in the canonical splice acceptor site of exon 6 in *NSUN2*, a conserved RNA methyltransferase, was identified in all three children [8]. Lastly Yue [9] found compound heterozygous mutations in the nuclear ligase gene *LIG4* in the sister from a pair of siblings previously reported with Dubowitz syndrome [2], [10] who are also investigated in this report (Patient 2).

We clinically characterized and performed exome sequencing and copy-number analysis on three individuals with Dubowitz syndrome: the pair of previously reported siblings (Patients 1 and 2) [2], [9], [10] plus an unpublished patient (Patient 3). Given the phenotypic overlap of Patients 1 and 2 with dyskeratosis congenita, we sought and identified abnormalities in telomere length and radiosensitivity [11], which prompted similar investigations in Patient 3. Our findings underscore the genetic and phenotypic heterogeneity of Dubowitz syndrome.

## Patient Descriptions

***Patients 1*** and ***2*** were participants in a longitudinal cohort study approved by the Institutional Review Board of the National Cancer Institute (NCI) entitled “Etiologic Investigation of Cancer Susceptibility in Inherited Bone Marrow Failure Syndromes” (NCI 02-C-0052). Patient 1 (NCI unique patient identifier 224-2) provided written informed consent and underwent detailed evaluation at the NIH Clinical Center. Patient 2 (NCI unique patient identifier 224-1) was deceased at the time of this study, but had been enrolled in the study prior to her death. Her *LIG4* mutation has been recently reported [9]. She was not evaluated at the NIH Clinical Center. Patient 3 was a participant in a study approved by the Institutional Review Board at the National Human Genome Research Institute entitled “Whole Genome Medical Sequencing for Gene Discovery” (10-HG-0065) and was evaluated at the NIH Clinical Center. His parents provided written informed consent to participate in the study. Patient 1 gave written informed consent (as outlined in the PLOS consent form) to publish these case details. The parents of patient 3 gave written informed consent (as outlined in the PLOS consent form) to publish their son's case details.

Patients 1 and 2 were originally reported in 1973 in a case series (as “Case 2” and “Case 1,” respectively) characterizing clinical features of Dubowitz syndrome [2]. The siblings were followed up in a short report describing aplastic anemia in Patient 2 (“Proposita”) and hematologic abnormalities in Patient 1 (“Brother”) [10]. No photographs have been published of the siblings. In brief, Patient 1 is a 41-year-old male with metabolic syndrome, panic attacks and anxiety, primary hypogonadism, learning disabilities, and dysmorphic features. He was born via vaginal delivery following a full term gestation (length was 43 cm (<5^th^ centile), weight was 2.4 kg (<5^th^ centile), occipitofrontal circumference (OFC) was 38.5 cm (<3^rd^ centile)) to a 19-year-old mother and 21-year-old father not known to be consanguineous. He was developmentally normal in infancy. He was diagnosed with Dubowitz syndrome at eleven months in the 1973 report [2], based upon minimal eczema, hyperactivity, characteristic high-pitched voice and appearance: small size, microcephaly, downslanted palpebral fissures, bilateral epicanthal folds, bulbous nose, micrognathia and mild cutaneous syndactyly of toes 2, 3, and 4. At an evaluation at 12 years of age described in the follow-up 1985 report [10], he had experienced multiple episodes of bilateral otitis media, small conical teeth, and continued hyperactivity. There was no history of bruising, lymphadenopathy, or hepatosplenomegaly; bone marrow biopsy showed spotty hypoplasia. There were no abnormalities on skeletal survey or excretory urogram. There was a normal 46,XY karyotype from peripheral blood, and a chromosomal breakage rate of 3%; on clastogenic breakage studies there were no spontaneous breaks and a diepoxybutane-induced rate of 0.08 breaks per cell. Hematologic indices showed leukopenia (WBC 2.9 K/µl), macrocytosis (MCV 103 fL), paradoxical reticulocytosis (reticulocyte 4.7%) without anemia (Hgb 11.6 gm/dL) and elevated fetal hemoglobin (10.0%).

His interval history was limited. At the time of NIH evaluation, he noted one-flight dyspnea on exertion, and chronic neuropathic pain in his legs for which he took naproxen twice a day. He denied tobacco or illicit drug exposure, and was a social drinker. There was no known history of potential toxic exposures to radiation, industrial chemicals or heavy metals. Details regarding his sister (Patient 2) are described below. His mother died at 52 years from heart failure. He was estranged from his father. His sister, Patient 2, was the only other family member with Dubowitz syndrome.

**Figure 1:**
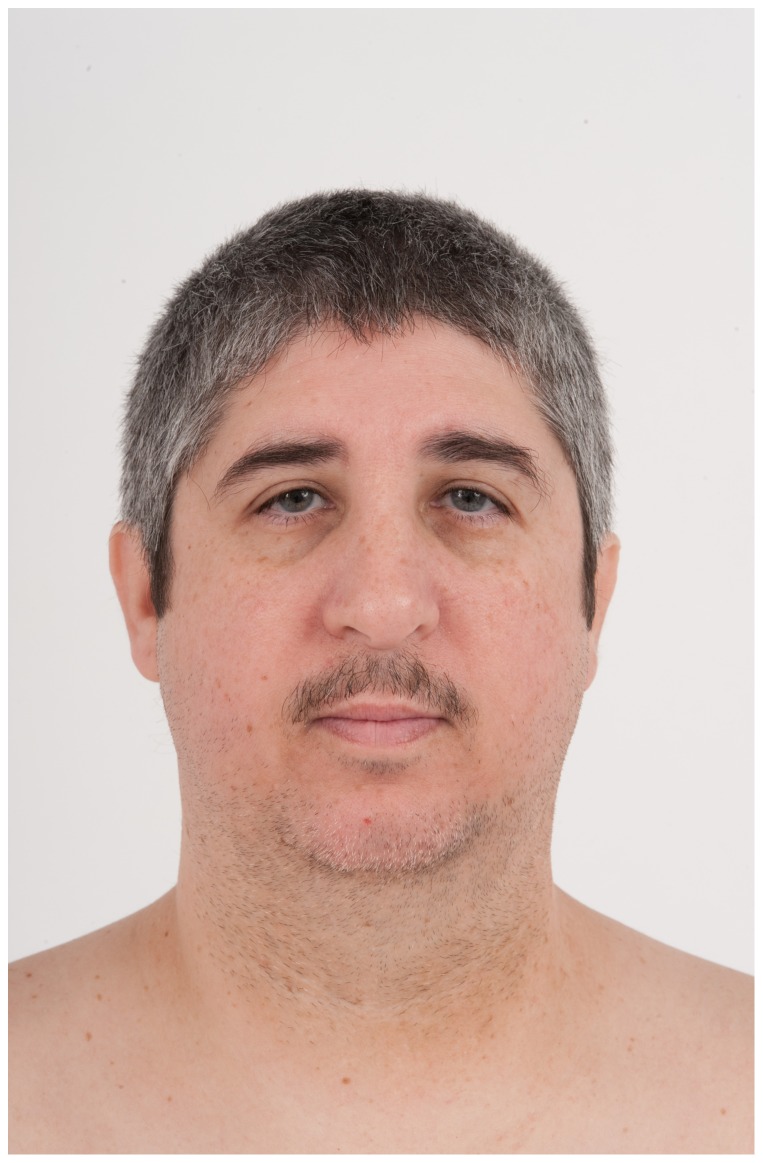
Frontal view of Patient 1 at age 41 years. He was originally diagnosed with Dubowitz syndrome (Opitz *et al*, 1973; Walters and Desposito, 1985). This is the first published photograph of this seminal patient. Exome sequencing identified compound heterozygote mutations in *LIG4*, confirming the formal diagnosis of *LIG4* syndrome. Dysmorphic features include microcephaly (<3^rd^ centile), bilateral downslanted palpebral fissures, low anterior hairline, and a long tubular nose with prominent bulbar tip.

**Figure 2:**
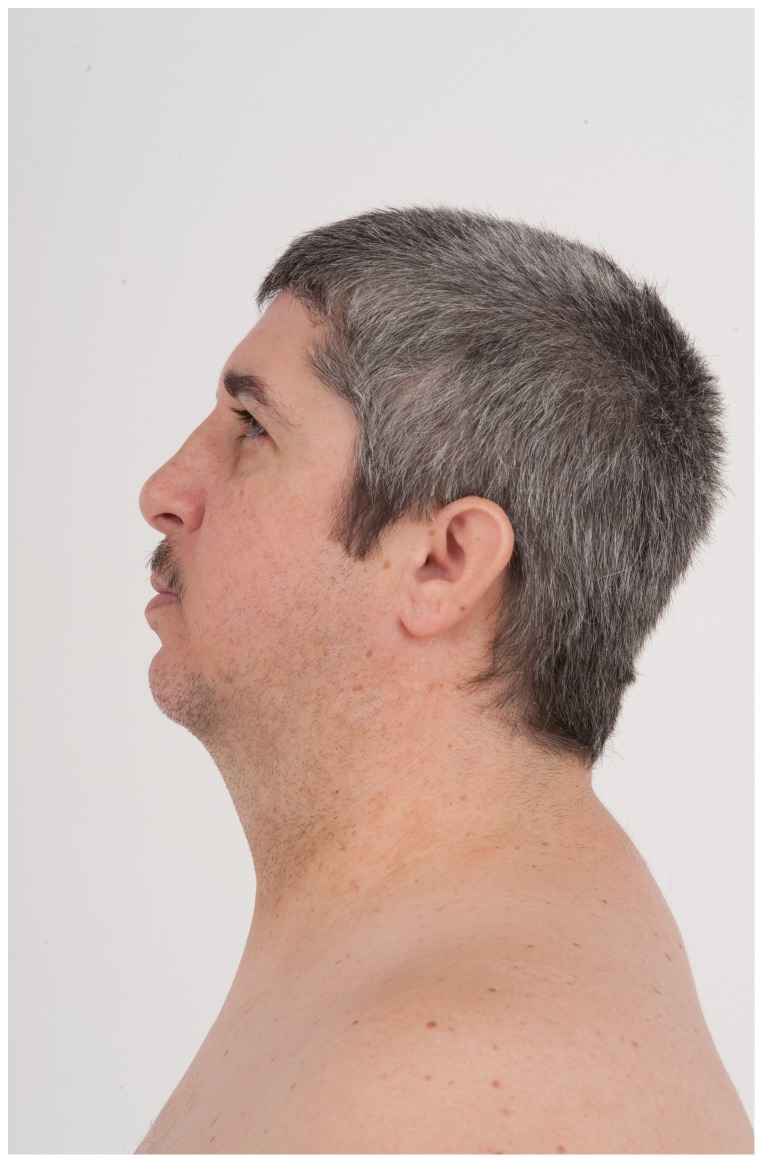
Profile view of Patient 1 at age 41 years. Dysmorphic features include a low posterior hairline, small (<−2 standard deviation), low-set ears and a high nasal bridge.

At age 41 years, Patient 1′s height was 157.2 cm (<5^th^ centile), weight was 74.1 kg (BMI 29 m/kg^2^), and his OFC was 52 cm (<3^rd^ centile, corrected for gender and height). He was a white male with an eunuchoid appearance, central obesity, a full head of graying hair, low anterior/posterior hairlines, and a clockwise hair whorl at the vertex. His ears were somewhat low-set and small (ear length 5.0 cm, <−2.0 SD) with no pits, tags or unusual rotation. There were bilaterally downslanted palpebral fissures; palpebral fissure length was 3.0 cm (normal). His interpupillary distance was 5.3 cm (3^rd^–25^th^ centile). There were neither ptosis nor epicanthal folds. His nose was long and somewhat tubular with a prominent bulb at the tip, and a high nasal bridge. He had a small mouth (Figures 1 and 2). Cardiopulmonary exam was normal. He had inverted nipples bilaterally and gynecomastia. His abdomen was obese with striae and a small midline umbilical hernia. His hands were short with tapered fingers; his nails appeared normal. There was mild 2–3 cutaneous syndactyly bilaterally. The hand and foot lengths were all <3^rd^ centile: right hand (15.5 cm), left hand (16.0 cm), right palm (9.0 cm), left palm (9.5 cm), right middle finger (6.5 cm), left middle finger (6.5 cm), right foot (22 cm) and left foot (22 cm). There were small (<5 ml) bilaterally descended testes, a small-uncorrected hypospadias and a micropenis. He was Tanner stage V. Neurologic exam was unremarkable except for diminished reflexes bilaterally.

Extensive laboratory testing and clinical evaluation showed: 1) normal cranial MRI, with a small pituitary; 2) extensive degenerative joint disease at L3–L4 on spine MRI; 3) metabolic syndrome; 4) primary hypogonadism (total testosterone <20 ng/dL); 5) a small diverticulum or aneurysm at the cardiac apex of uncertain significance; 6) a mixed obstructive/restrictive pattern on pulmonary function tests with a normal chest CT scan; 7) sclerotic and lucent features on right femoral head compatible with avascular necrosis; 8) normal 46,XY male karyotype; and 9) hypocellular bone marrow with trilineage hypoplasia and anemia (Hgb 12.2 g/dL; RDW 15.8%; MCV 96.5 fL; ferritin 171 mcg/L; fetal Hgb 8.9%). Total cholesterol 205 mg/dL; triglycerides 143 mg/dL; HDL cholesterol 40 mg/dL; LDL cholesterol 136 mg/dL. Quantitative serum immunoglobulins: IgG 743 (642–1730 mg/dL), IgA 184 (91–499 mg/dL), IgM 111 (34–342 mg/dL). Lymphocyte phenotyping: CD3 count 547 (615–2348/uL), CD4/CD3 count 269 (334–1556/uL), CD8/CD3 count 303 (149–787/uL) and CD19 count 5 (81–493/uL).

Patient 2 is the younger sister of Patient 1; she died at age 35 years from complications of squamous cell cancer of the anus. As described in the 1973 Opitz report [2], she was born at 32 weeks following a normal pregnancy with birth weight 1.6 kg and birth length 40.5 cm; she was in the newborn nursery for one month. She was hospitalized three times in the first seven months for respiratory difficulties. Examination at 7.5 months showed a small infant with an unusual high-pitched voice, length 57 cm, weight 4.5 kg and OFC 35.5 cm (all <3^rd^ centile). There was apparent microcephaly and brachycephaly with closed fontanels, anteverted nares, a broad nasal bridge, micrognathia, fine/sparse hair and minimal cutaneous syndactyly of toes 2, 3, and 4 bilaterally. No photographs were available. The follow-up 1985 report [10] described her at age 10 years, with height 119 cm (<3^rd^ centile) and OFC 48 cm (<3^rd^ centile). She had cognitive delay and required special education. There was easy bruising and pallor with pancytopenia (Hgb 6.9 gm/dL; MCV 104 fL; WBC 2.1 K/µl; platelets 10 K/µl; fetal hemoglobin 7.3%). She was transfusion-dependent. Physical exam showed short palpebral fissures, epicanthal folds, broad nose, micrognathia and 2–3 cutaneous syndactyly. The facial eczema present in early childhood had resolved. She still had a distinctive high-pitched, hoarse voice. Bone marrow biopsy showed severe hypoplasia and normal female 46,XX karyotype from peripheral blood. Her mean spontaneous chromosome breakage rate per cell was 0.04 (normal 0–0.05) and a diepoxybutane-induced breakage rate of 0.12 breaks per cell (normal 0–0.10). Treatment was started with oxymetholone. This treatment resulted in improved counts; no additional transfusions were needed. This therapy was continued until approximately age 24 years. Her growth was noted to have stopped shortly after starting the oxymetholone. She was an occasional smoker.

At age 34 years, she presented with pancytopenia and bright red blood per rectum; colonoscopy showed a 5-cm ulcerative mass in the posterior rectum just inside the anal verge. Biopsy of the mass was compatible with locally advanced squamous cell carcinoma of the anus (clinical T2/3 N0 M0 stage II). Anal brushings were positive for high-risk human papillomavirus (HPV) DNA (genotypes 16, 18, 31, 33, 35, 39, 45, 51, 52, 56, 58, 59 and 68). There was also a history of rectal warts. Testing for HIV 1 and 2 antibodies was negative. A metastatic work-up was negative. By this time, she had been diagnosed with diabetes and dyslipidemia. The oxymetholone had been restarted. Repeat bone marrow biopsy showed 1% cellularity consistent with aplastic anemia. She was treated with radiation therapy only but developed extensive radiation dermatitis and perirectal desquamation after receiving 2000 cGy to the anus. This was slow to heal and she had persistent peri-rectal pain, rectal bleeding, progressive weight loss, fatigue and bilateral lower extremity weakness. At first the anal mass was noted to shrink modestly. However three months after cessation of radiation therapy, a 3.4 cm mass in the posterolateral aspect of the bladder and sigmoid colon - consistent with tumor recurrence - was noted on CT scan. She continued to have complications from radiation proctitis and died following a lower gastrointestinal bleed at age 35 years.

***Patient 3*** was a 16-year-old boy diagnosed with Dubowitz syndrome at age 10 years based on developmental delay, short stature, IUGR and dysmorphic features. He was born to a 30-year-old woman (father was 36 years old) by spontaneous vaginal delivery at 36 weeks; pregnancy was complicated by fetal liver calcifications and oligohydraminos. His birth weight was 4 pounds, 0 ounces (<10^th^ centile; 50^th^ centile for 33-week gestation) birth length was 16 inches (<10^th^ centile; 50^th^ centile for 30-week gestation) and birth OFC was 30 cm (<<1^st^ centile). His eyebrows and eyelashes were noted to be absent at birth. He was in the NICU for six days due to gastroesophageal reflux and poor feeding. A peripheral blood karyotype at birth showed 46,XY. Subtelomeric chromosome deletion testing around age 6 years was normal. Numerous diagnoses have been considered, including progeria, autism, uniparental disomy, and *cri du chat*. Prior to evaluation at the NIH, his past medical history included: 1) partial anal obstruction, repaired surgically at 2.5 months of age; 2) reflux disease and poor feeding, managed with a Nissen fundoplication at 10 months (and revision at 4 years), cisapride (discontinued due to prolonged QTc interval and premature ventricular complexes) and gastrojejunal tube; 3) chronic constipation and encopresis; 4) growth hormone deficiency with replacement from age 20 months to 13 years (discontinued due to worsening kyphosis and scoliosis); 5) developmental delay (12 word vocabulary at 15 years); 6) frequent otitis media with tympanostomy tubes; 7) left eye strabismus, corrected surgically at age 5 years; 8) incidental patent foramen ovale; 9) bladder spasm; 10) elevated triglycerides, (although this was present is both parents and several paternal relatives and is likely to be unrelated to his primary condition); 11) short stature (at age 5.5 years, his height was 102.2 (<5^th^ centile or 50^th^ centile for a 4-year-old) and underweight 17.7 kg (∼25^th^ centile); OFC 49 cm (∼10^th^ centile)) and; 12) small focal heterotopias along the lateral ventricles, normally formed corpus callosum, brainstem, cerebellum and vermis, with a myelination delay of 4–5 months, on cranial MRI done at age 4 months. At the time of the NIH evaluation, he was taking lansoprazole, a multivitamin, and calcium. He had no known allergies. His primary teeth appeared at age 2 years. Anterior fontanel closure occurred at age 2 years. He learned to walk at 20 months. He was of European ancestry. Family history was notable for a healthy 19-year-old sister. His parents were healthy and non-consanguineous. His maternal grandmother's sister had a daughter with two children, each with significant health issues: a 16-year-old male with history of imperforate anus and developmental delay, and a 13 year-old daughter with hydrocephalus and developmental delay.

**Figure 3:**
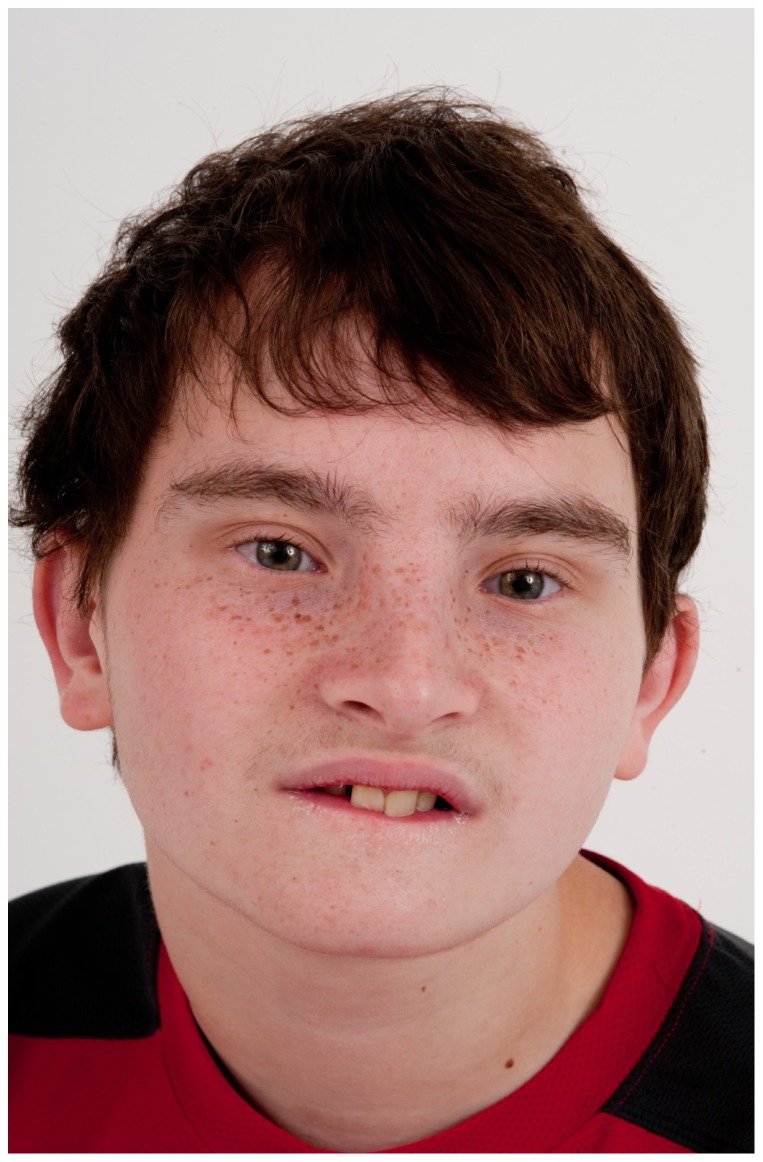
Frontal view of Patient 3 at age 15 years. He was originally diagnosed with Dubowitz syndrome and was determined to harbor a 3.89 Mb deletion on chromosome 17q24.2. Dysmorphic features include bilateral upslanted palpebral fissures with apparent telecanthus, broad nasal bridge and a bulbous nose.

**Figure 4:**
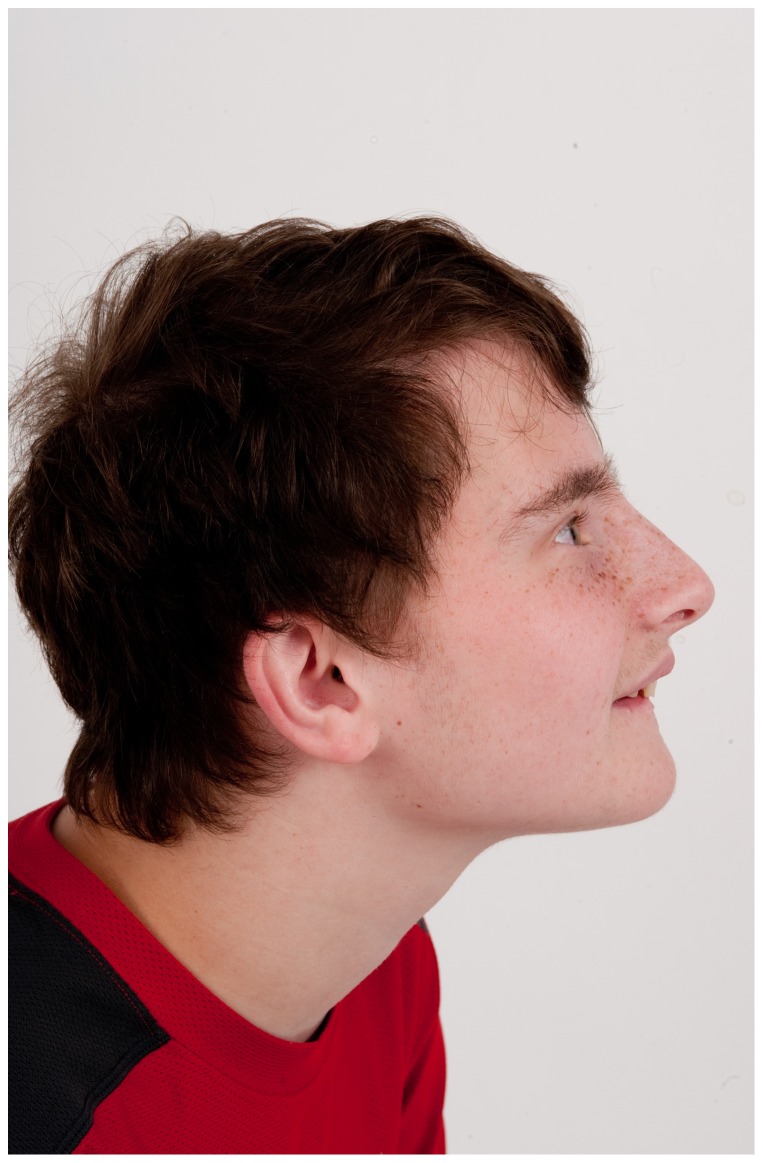
Profile view of Patient 3 at age 15 years. Dysmorphic features include low set ears.

**Figure 5:**
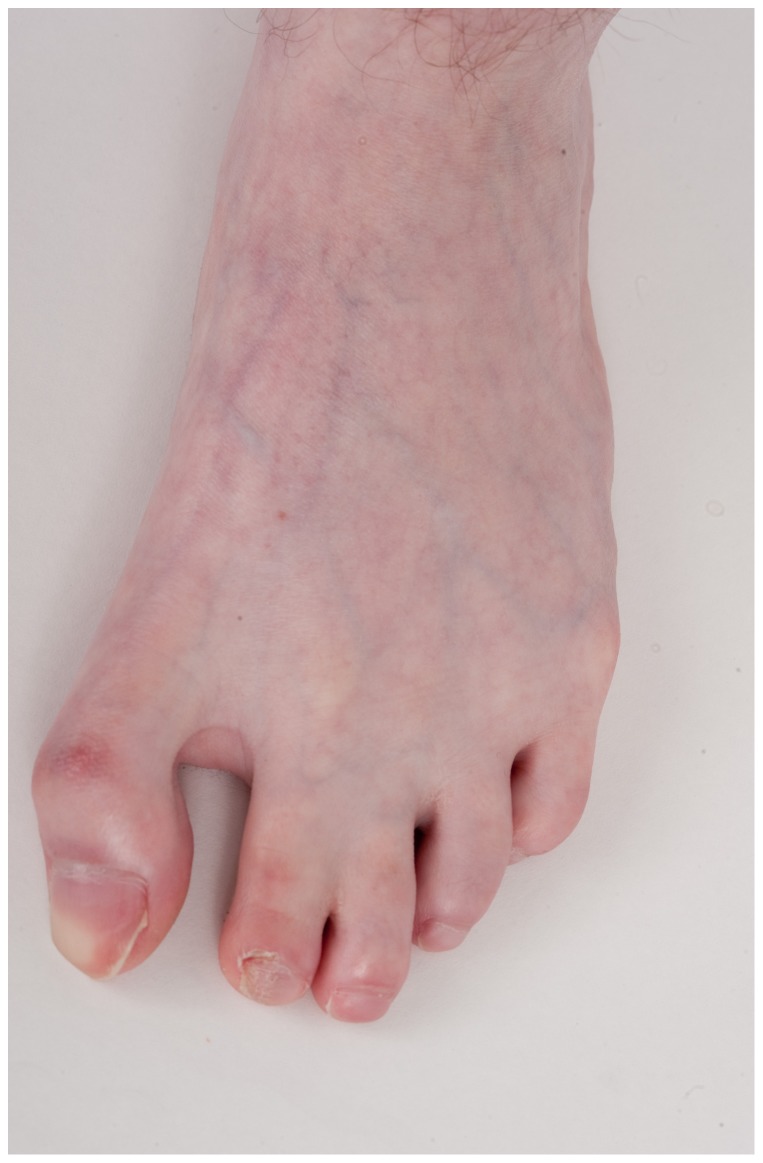
2–3 cutaneous syndactyly and appearance of cutis marmorata telangiectasia congenita in Patient 3.

He was initially evaluated at age 15 years at the NIH. His height was 142.5 cm (<3^rd^ centile; 50^th^ centile for 11 years), weight was 39.4 kg (<3^rd^ centile; 50^th^ centile for 11.5 years), and OFC was 54 cm (∼25^th^ centile). He appeared anxious with near-continuous teeth grinding and unintelligible speech. There were bilaterally upslanted palpebral fissures with apparent telecanthus. His outer canthal distance was 9.5 cm, inner canthal distance 3.7 cm, inner pupillary distance 6.25 cm (∼90^th^ centile). The palpebral fissures lengths were 2.9 cm (−1 standard deviation). His eyebrows were full with no arching or synophrys. He had a broad nasal bridge. He had a bulbous nose. There was freckling across his nose and under his eyes. His ears were normally formed but somewhat low-set and posteriorly rotated, with a length of 5.5 cm (−1.5 standard deviations); see Figures 3 and 4. Overall, his skin had the appearance of cutis marmorata telangiectasia congenita (Figure 5). Cardiopulmonary exam was normal. The abdominal exam was normal except for a left upper quadrant surgical scar and PEG tube scar. He was tanner IV with bilaterally descended testes and a sacral dimple. He had tapered fingers and a slight resting hand tremor. Third finger length bilaterally was 6 cm (<3^rd^ centile; 50^th^ centile for 8-year-old); right hand length was 14 cm (<3^rd^ centile; 50^th^ centile for 7.5 year old) and left hand length was 15 cm (<3^rd^ centile; 50^th^ centile for 9-year-old). He had bilateral 2–3 cutaneous toe syndactyly. Ophthalmology consultation showed mild intermittent exotropia. An abdominal ultrasound was unremarkable. At a chronological age of 14 years, 11 months, a bone age from the left hand and wrist showed ossification was consistent with the male standard of 17 years. A scoliosis series showed mild-to-moderate thoracic kyphosis. Cholesterol testing showed total cholesterol 216 mg/dL, triglycerides 375 mg/dL, HDL 35 mg/dL and LDL 106 mg/dL. A CBC showed WBC 13.85 K/µl, Hgb 14.9 g/dL, MCV 92.4 fL, platelets 267 K/µl.

Brief follow-up evaluation at age 16 years at the NIH showed a height of 141.3 cm (<3^rd^ centile; 50^th^ centile for 10.5 years), and weight of 42.4 kg (<3^rd^ centile; 50^th^ centile for 11.5 years). A focused evaluation for Carney complex showed a normal echocardiogram with no evidence of atrial myxoma, insulin-like growth factor-1 368 ng/mL (193–731 ng/mL), serum cortisol 21.1 mcg/dL (5.0–25.0 mcg/dL), adrenocorticotropic hormone 70.6 pg/mL (0–46.0 pg/mL) and growth hormone 0.11 ng/mL (0–0.80 ng/mL).

## Results

### Lymphocyte telomere length as a clue to etiology

**Figure 6:**
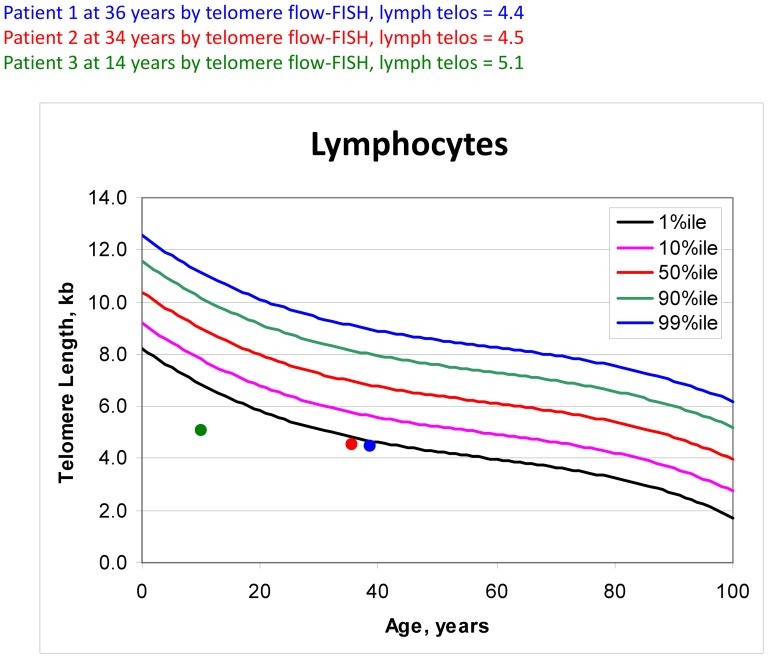
Median telomere lengths in lymphocytes, as determined by flow cytometry with fluorescent *in situ* hybridization (flow FISH). Patients 1 (two time points), 2 and 3 are plotted against population norms. yrs  =  years; MTL  =  median telomere length; kb  =  kilobase

Figure 6 shows the lymphocyte telomere lengths of these patients, all three of whom had telomeres ≤1^st^ centile for their age. This suggested that a defect in telomere maintenance could be involved in the etiology of this disease. Patients with dyskeratosis congenita, the prototypical telomere biology disorder, have leukocyte telomeres <1^st^ centile for their age, germline mutations in key telomere biology genes, bone marrow failure and multiple other findings, including the diagnostic clinical triad of nail dysplasia, lacy skin pigmentation, and oral leukoplakia [12].

### Colony survival assays showed increased sensitivity to ionizing radiation in Patients 1, 2 and 3

**Figure 7:**
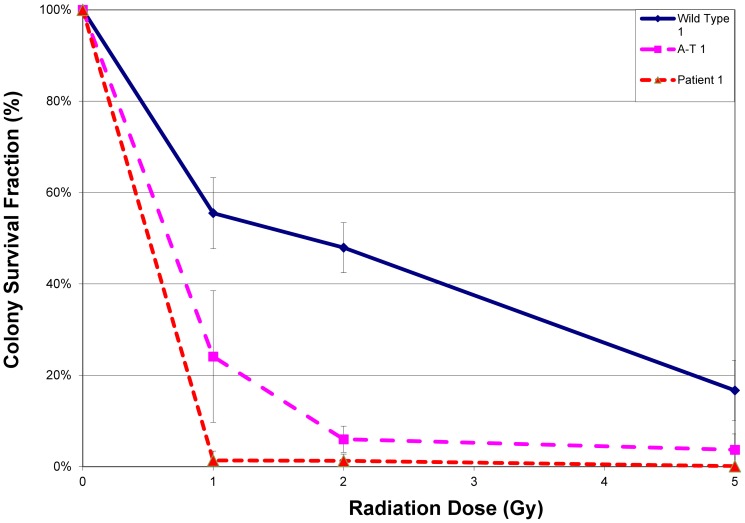
Colony survival assay demonstrates cellular radiosensitivity in Patient 1. Fibroblasts from Patient 1 were highly sensitive to increasing doses of ionizing radiation. Experiments were performed in triplicate with the average of three experiments shown. WT  =  wild type; A-T  =  ataxia-telangiectasia control.

**Figure 8:**
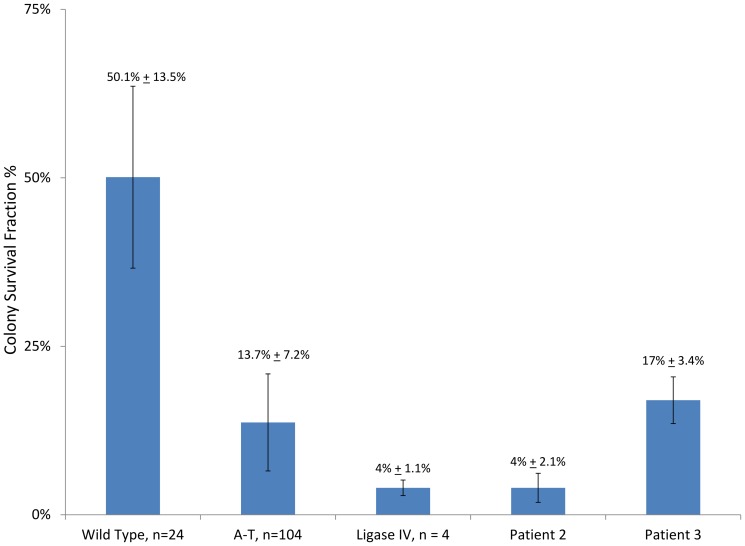
Colony survival assay demonstrates cellular radiosensitivity in Patients 2 and 3. Lymphoblastoid cell lines from Patients 2 and 3 were radiosensitive, as compared to controls. Experiments were performed in triplicate with the average of three experiments shown. WT  =  wild type; A-T  =  ataxia-telangiectasia control. Ligase IV  =  Ligase IV-deficient control.

Colony survival assay (CSA) showed diminished survival of a fibroblast cell line from Patient 1 (Figure 7), and LCLs from Patient 2 and 3 (Figure 8). The typical range for healthy control cell lines is 50.1% +/−13.5%. Individuals with known *ATM* mutations have CSA values of 13.1% +/−7.2% [13].

### Neutral comet assay compatible with a DNA double-strand break repair disorder

**Figure 9:**
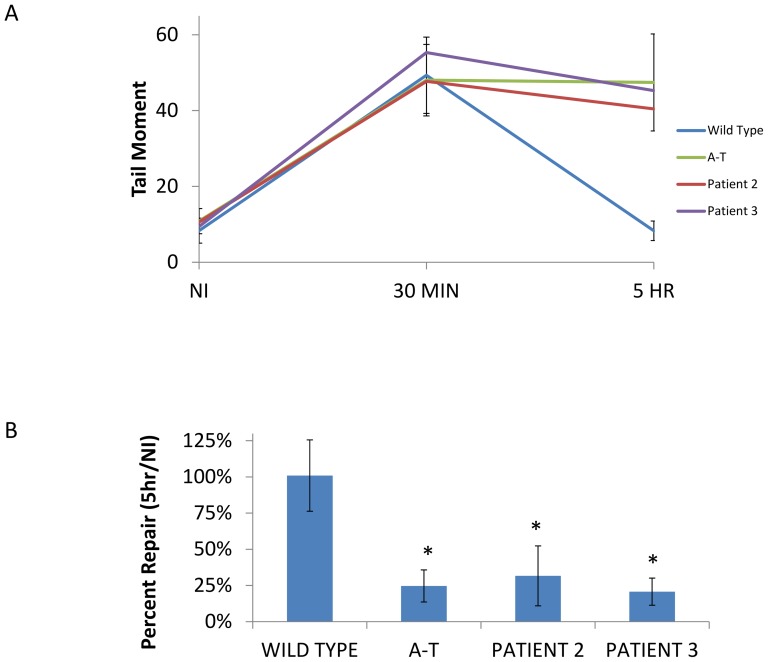
Neutral comet assay compatible with a DNA double-strand break repair disorder. (a). Tail moment of WT, A-T and patient cells at 0, 30 minutes and 5 hours post-irradiation. All cells show damage at 30 minutes post-irradiation. WT returns to near baseline levels after 5 hours, while A-T and patient cells show long comet tails after 5 hours, indicating significant levels of unrepaired DNA. (b). Ratio of unrepaired DNA at 5/0 hours. A-T as well as Patient 2 and 3 cells show statistically significantly lower levels of repair at 5 hours post-irradiation compared to wild type cells (* p<0.05). Experiments were performed in triplicate with the average of three experiments shown. WT  =  wild type; A-T  =  ataxia-telangiectasia control.

The neutral comet assay measures the level of unrepaired DNA in a cell [14]–[16]. Recent studies have shown a correlation between double-strand DNA post-irradiation damage, as measured by NCA, and the colony survival assay [14]. Longer, undamaged DNA fragments will remain predominantly in the “head” of the comet, while smaller, heavily damaged DNA fragments will migrate more quickly and form the tail of the comet. The average tail moment at 5 hours post-irradiation is divided by the average tail moment of non-irradiated cells to determine the percent DNA repair. Wild type cells with normal DNA double-strand-break repair abilities will re-ligate most breaks within 5 hours while cells with deficient repair mechanisms will have a much lower percent DNA repair. The results from both patients 2 and 3 show large amounts of unrepaired DNA at 5 hours post-irradiation (Figure 9). Their percent repair is significantly less than wild type cells and is similar to the repair efficiency of *ATM*-deficient cells. These results, along with the concordant, abnormal CSA scores in both patients are compatible with a DNA double-strand break repair disorder.

### Patients 1 and 2 harbored compound heterozygous mutations in LIG4

**Figure 10:**
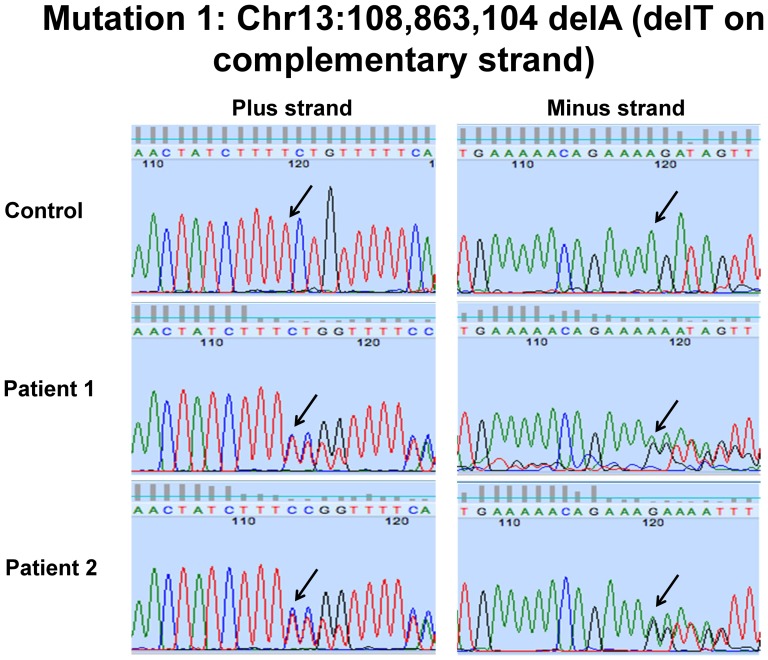
Sanger sequencing of *LIG4* c.613delT variant in Patients 1 and 2.

**Figure 11:**
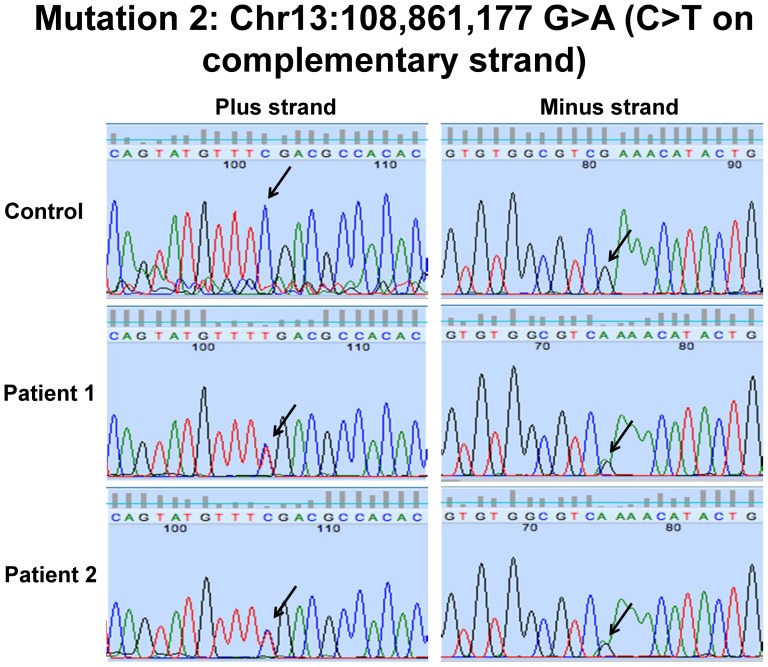
Sanger sequencing of *LIG4* c.2440C>T variant in Patients 1 and 2.

**Figure 12:**
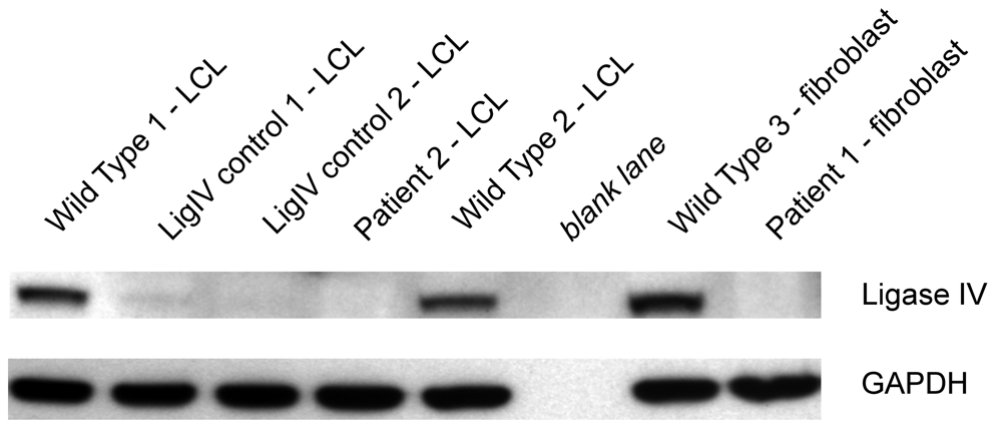
Western blot of whole cell lysates from Patient 1 fibroblasts and Patient 2 lymphblastoid cell lines. Patients 1 and 2 show an absence of ligase IV protein. Lanes were loaded with 50 µg protein/lane. LCL  =  lymphoblastoid cell line;

Exome sequencing with Sanger sequencing verification showed a known rare nonsense substitution predicted to result in a premature stop codon (rs104894419, NM_002312.3:c.2440C>T, p.Arg814X, MAF: 0.0002) and a single base pair deletion predicted a frameshift and premature stop codon (NM_002312.3:c.613delT; p.Ser205Leufs*29) (Figures 10 and 11). The rs104894419 variant had a MAF of 0.0002 in the ESP European-American cohort. The frameshift mutation had not been reported in 1000 Genomes, ESP or the ClinSeq databases. The two variants were in *trans*, consistent with autosomal recessive inheritance (Table S2). Western blotting showed absence of ligase IV band in both Patients 1 and 2 (Figure 12).

### Patient 3 harbored a ∼3.89 Mb deletion at chromosome 17q24.2

Analysis of high-density array SNP genotyping showed a *de novo* ∼3.89 Mb interstitial deletion at chromosome 17q24.2 (chr 17: 62,068,463–65,963,102, hg18). The deletion was confirmed by array-based comparative genomic hybridization using a custom designed oligonucleotide microarray (GeneDx, Gaithersburg, MD). The deletion encompasses 26 annotated RefSeq genes (genome.ucsc.edu). There are copy number variations (CNV) in normal controls within this interval on chromosome 17, but none overlapping all 27 genes (Database of Genomic Variants; http://projects.tcag.ca/variation/). In particular, deletions of *PSMD12*, *AMZ2*, and *PRKAR1A* have not been reported in control populations and reported deletions in *ARSG* and *MAP2K6* do not involve obligate exonic regions. No other significant *de novo* copy-number changes were observed elsewhere in the genome of Patient 3. Array SNP genotyping in Patients 1 and 2 was consistent with a full-sibling relationship (Figure S1). The siblings both harbored a ∼1.5 Mb CNV gain at chr13: 94,700,000–94,850,000 (hg18) encompassing the 5′ end of the ATP-binding cassette transporter *ABCC4*; CNVs in this region are common (Database of Genomic Variants; http://projects.tcag.ca/variation/).

### Analysis of exome data from Patient 3

Exome sequence data were generated for the proband and both parents. A total of 146,181 quality variants (MPG ≥10 and MPG/coverage ≥0.5) were identified in the proband. Filtering for quality across all three individuals in the trio reduced the variant count to 126,876. The analysis was restricted to variants predicted to result in non-synonymous, nonsense, frame shifting, or splice site alterations. Three parallel filtering strategies based on inheritance were employed to generate a list of potential causative variants: dominant *de novo* alteration (variant present in proband but absent in both parents), recessive homozygous alteration (variant present in the homozygous state in proband and heterozygous state in both parents), or recessive compound heterozygous alterations (two or more variants in a single gene present in proband and at least one variant present in each parent). For the dominant *de novo* filter, variants present in a control set of 951 individuals were excluded. For both recessive filters, variants were excluded if they were found to be homozygous in a control set of 951 individuals or had a reported SNP frequency of 0.15 or greater. A total of 34 candidate variants were identified: 3 potential *de novo* dominant variants, 4 potential homozygous recessive variants, and 27 potential compound heterozygous variants in 9 genes (Table S3). In patient 3′s *de novo* chromosome 17 ∼3.89 Mb deletion identified by SNP array analysis (chromosome 17: 62,068,463–65,963,102 (NCBI36/hg18)), eleven sequence variants were identified (Table S4). All eleven variants were found to be homozygous in the control dataset of 951 variants and were determined unlikely to be causative. Parentage was confirmed by the thousands of variants in common between the child and his parents on the exome trio analysis.

## Discussion

We report the genetic etiology of three individuals diagnosed clinically with Dubowitz syndrome. Patients 1 and 2 were previously-reported siblings [2], [9], [10]. All three patients shared numerous features of Dubowitz syndrome [3] including IUGR, short stature, reduced head circumference, cognitive delay, facial gestalt and a variety of other dysmorphic findings. In addition to a Dubowitz syndrome diagnosis, the phenotypes of Patients 1 and 2 overlapped with the clinical spectrum of dyskeratosis congenita; an evaluation for this disorder prompted the discovery of markedly shortened telomere length [11] and abnormal colony survival and neutral comet assays, but no mutations in the known dyskeratosis congenita genes *TERT*, *TERC*, *DKC1*, *TINF2*, *WRAP53, NOP10, NHP2, CRC1* or *RTEL1*. Forty additional telomere biology genes were normal upon careful evaluation of exome sequencing data. Similarly shortened telomeres and abnormal CSA and NCA results in Patient 3 initially suggested that these tests might be useful in characterizing patients with a Dubowitz diagnosis. However the subsequent identification of *LIG4* mutations in Patients 1 and 2 following exome sequencing and the identification of a chromosome 17q24.2 deletion in Patient 3 (spanning the known Carney complex gene *PRKAR1A*) served to underscore the genetic heterogeneity of the disorder. Our findings suggest that what is currently termed Dubowitz syndrome is a syndrome complex or spectrum comprised of multiple genetically distinct but phenotypically overlapping disorders.

The DNA ligase IV deficiency syndrome is a rare autosomal recessive disorder that arises from mutations in the DNA ligase IV (*LIG4*) gene. The *LIG4* gene product ligase IV is a nuclear ligase that is a key component of the nonhomologous end-joining (NHEJ) machinery, a major DNA repair pathway for remediating double-strand breaks in mammalian cells following DNA damage as well as during class switching and V(DJ) recombination [17]. Recently, *LIG4* mutations were independently identified in Patient 2 by another group [9], based on a candidate gene strategy that arose from the striking radiosensitivity of the patient. Our findings are concordant with this previous report. Not surprisingly, her brother Patient 1 was also a compound heterozygote (as determined by phase analysis; see Figure S1) for the same *LIG4* mutations. He features similar *in vitro* radiosensitivity.

Although *LIG4* mutations in this family have been described previously [9], we report, for the first time, shortened telomeres in ligase IV deficiency. This observation may be diagnostically relevant. Telomere length, as determined by clinical testing using flow-FISH, is useful in the diagnosis of dyskeratosis congenita, a heterogeneous genetic disorder with mucocutaneous abnormalities, bone marrow failure and tumor predisposition. [12]. Terminal telomeric DNA is maintained by a highly-regulated, complex set of proteins including components of the telomerase holoenzyme, the shelterin complex, and specific trafficking and capping proteins; mutations in one of nine different telomere biology genes can cause dyskeratosis congenital [18]. Telomere length <1^st^ centile in any four of six leukocyte subsets (lymphocytes, granulocytes, CD45RA-positive, CD45RA-negative, CD20-positive and CD57-positive) or three of the five subsets if granulocytes are excluded is consistent with the diagnosis of dyskeratosis congenita [19]. Patients 1 and 2 had telomere lengths less than the 10^th^ centile in all leukocyte subsets and many were <1^st^ centile. Ligase IV, which primarily has a role in classic NHEJ, is not known to be associated with the telomerase enzyme complex; however, given its key role in DNA repair, ligase IV defects could affect telomeres [20]. Studies of yeast ligase IV show that it is not critical in telomere length homeostasis [21] or telomere fusions [22]. The literature is inconsistent with one study suggesting an increased rate of telomere shortening in *LIG4*-deficient fibroblast cell lines 180BR [23] and 411BR [24] although this was not observed in *LIG4* heterozygote cells [25]. This finding contradicted an earlier study that found normal telomere length in 180BR cells [26]. These limited data in yeast and human cell lines are contradictory and difficult to interpret. Our finding of shortened telomeres in peripheral leukocytes subsets from Patients 1 and 2, if confirmed in other *LIG4*-deficient individuals, will likely be clinically useful and may suggest a previously unrecognized role for ligase IV in telomere homeostasis.

There is considerable overlap in clinical phenotype, including dysmorphic features, radiosensitivity, immunodeficiency, risk of bone marrow failure and leukemia predisposition, between *LIG4* syndrome and other disorders of DNA repair, including Nijmegen breakage syndrome, Fanconi anemia, and Seckel syndrome [17], [27]. The diagnosis of Dubowitz syndrome has been considered in other patients later determined to have *LIG4* mutations [28]. *LIG4* mutations are rare; there are only 15 patients with detailed human phenotypes reported in the literature (Table S5) [23], [24], [27]–[36]. Including the two siblings reported here, there are four males, ten females and three with unknown gender. Most patients were recognized in early childhood, but some were diagnosed in early infancy [34], especially if an older sibling was known to be affected. The oldest surviving patients were siblings aged 46 and 48 years [24]. In *LIG4* deficiency, radiosensitivity is a universal feature *in vitro*, and can manifest clinically as a severe reaction to radiotherapy [23], [36], as was observed in Patient 2. There were four cancers in the literature: two patients developed T-cell acute lymphoblastic leukemia [23], [27], and two developed diffuse large B-cell lymphoma [31], [33]. Patient 2 reported here is the fifth case: she developed locally advanced squamous cell carcinoma (SCC) of the anus; anal brushings were positive for high-risk HPV-DNA genotypes and she had a history of smoking, both known risk factors [37], [38]. Although SCC is rare and predominantly affects individuals older than 50 years, the age of diagnosis in Patient 2 is not unusual, since SCC rate among white women from 1973-2000 aged 20–49 years was 0.55/100,000 [39]. If the etiology of her cancer cannot be specifically ascribed to her *LIG4* mutations, her short survival (less than one year after diagnosis) may be in part due to her poor therapeutic response to radiotherapy and the persistent complications related to her unusually severe radiation injury, which prevented administration of full therapeutic doses. By comparison, from 1973–2000, the 5-year survival for women ages 20–49 years with anal SCC was 72% [39].

The majority of published patients with *LIG4* mutations had significant clinical immunodeficiency, including a novel severe combined immunodeficiency (SCID) presentation requiring bone marrow transplant [35], although this might reflect a reporting bias (Table S5). Neither Patient 1 nor Patient 2 in this report had a history of significant, recurrent infections (other than childhood otitis media) despite hypocellular or acellular bone marrow. Despite the possibility of bone marrow transplants, immunodeficiency and an apparent increased risk of leukemia impose a high mortality in *LIG4* syndrome, with six patients alive, eight deceased and three with unknown status.

Most patients with *LIG4* mutations had dysmorphic features (“Seckel-like” facies, epicanthal folds, up- or down-slanted palpebral fissures), IUGR, microcephaly, short stature and developmental delay, although not all [23], [29], [31]. In older patients, endocrine problems were common: hypogonadism, hypothyroidism, amenorrhea, and diabetes (Table S5). There were four (28%) males among the 14 patients with known gender; of these, two (50%) had micropenis and/or hypospadias. There was no apparent genotype-phenotype correlation.

We also observed shortened telomere in Patient 3, who harbored a microdeletion spanning portions of chromosome 17q24.2–q24.3. The deletion affects 29 known genes or predicted genes, four known microRNAs and one known lincRNA. Genes in this region associated with human phenotypes include *PRKAR1A* (Carney complex) [40] and *KCNJ2* (Andersen/Andersen-Tawil syndrome) [41]. In addition, the deleted locus is associated with multiple distinct genomic disorders arising from copy-number variation: 17q24.2 syndrome [42], Carney Complex plus [43], 17q24.2–q24.3 microdeletion syndrome [44] and congenital generalized hypertrichosis terminalis (CGHT) with or without gingival hyperplasia [45]. The microdeletion in Patient 3 reported here encompasses *in their entirety* the deletions reported in Carney Complex plus [43], and CGHT [45]. It also spans portions of the deletions (including most of the “smallest region of overlap” (SRO)) found in four patients with the 17q24.2 syndrome [42] and 17q24.2–q24.3 microdeletion syndrome [44]. Exome sequencing of the non-deleted chromosome 17q24.2 in Patient 3 did not show any novel or rare variants (Table S4), making the presence of a point mutation in *trans* to the deletion unlikely.

Overall the best *phenotypic* match for Patient 3 is with the Carney Complex plus patient [43], who had few overt manifestations of Carney complex but had IUGR, feeding difficulties, global developmental delay (although not as severe), short stature (<1^st^ centile), microcephaly (<<3^rd^ centile), 2–3 cutaneous syndactyly, widely spaced eyes, freckling, broad nasal bridge and convex nasal ridge. The feeding difficulties, developmental and cognitive delay, mottled skin, drug-induced long QT syndrome, short stature, microcephaly, small ears, 2–3 cutaneous syndactyly and dysmorphic features, again with few overt features of Carney complex, in one of the reported patients with 17q24.2 syndrome [42] also overlap with those of Patient 3. Patient 3 had no evidence of hypertrichosis, although his deletion spanned *MAP2K6*, the gene hypothesized to underlie this phenotype [45].

Of the ten patients with a microdeletion or an inverted duplication in 17q24.2–q24.3 and detailed phenotype data [42]–[45], there were no reports of testing for shortened telomeres or clinical or *in vitro* radiosensitivity. The best candidate genes in the deleted interval to explain shortened telomeres or clinical or *in vitro* radiosensitivity are *HELZ*, a putative RNA helicase [46] and *KPNA2*, a member of the karyopherin α (importin α) family. *KPNA2* has a role in nucleocytoplasmic transport and is responsible for the import, via its nucleus localization sequence, of *NBN*, the DNA repair gene [47] mutated in Nijmegen breakage syndrome [48]. Repression of *KPNA2* expression causes reduction in radiation-induced nuclear focus accumulation and double-stranded DNA repair [49]. *HELZ* or *KPNA2* are deleted in all of the patients reported by Vergult [42] (17q24.2 microdeletion) and Blyth [43] (17q24.2–q24.3 microdeletion), but not those reported by Lestner [44] and Sun [45] (both 17q24.2–q24.3 microdeletions). The deletion of *KPNA2* and its association with *NBN* links Patient 3 to Patients 1 and 2 and the well-established phenotypic overlap of *LIG4* and Nijmegen breakage syndrome.

In summary, we report three patients originally diagnosed with the Dubowitz syndrome but who were instead determined to have *LIG4* mutations (Patients 1 and 2) or a deletion at chromosome 17q24.2–24.3 (Patient 3). (Of note, mutations in *LIG4* have been previously reported in Patient 2 [9].) We found shortened telomeres in these three patients, a novel finding in patients with mutations in *LIG4* and the chromosome 17q24.2–24.3 deletion; to confirm our observations, telomere length testing should be considered in both disorders. Ligase IV has not been previously implicated in telomere homeostasis. The telomere length assay in particular may prove useful in parsing the entities that currently constitute the Dubowitz syndrome diagnosis. Although radiosensitivity is well-recognized in mutation carriers of *LIG4*, it is a novel feature in deletions of chromosome 17q24.2–24.3. Our findings reflect the phenotypic and molecular heterogeneity of the Dubowitz syndrome diagnosis, as currently recognized. Taken together, our work and other reports on Dubowitz syndrome suggest that it is not a unitary disorder but instead a collection of phenotypically similar disorders. As a syndromic entity, the Dubowitz diagnosis will need continual re-evaluation and re-definition as its constituent phenotypes are determined.

## Materials and Methods

### Measurement of telomere length

Telomere length was determined in leukocyte subsets by flow cytometry with fluorescent *in situ* hybridization (flow FISH) using previously-described methods (Repeat Diagnostics, North Vancouver, BC, Canada) [50].

### Exome sequencing and analysis of variants

Peripheral blood lymphocyte DNA from Patients 1 and 2 was subject to exome capture, sequencing, and variant calling as described [51]. Dubowitz syndrome is rare; an autosomal recessive pattern of inheritance is most likely, although we also considered an autosomal dominant model of inheritance. Variants were considered as etiologic candidates if they 1) were shared between the siblings, 2) were detected in at least 10 uniquely mapped reads, 3) had a sequencing base quality value of at least 15, 4) were not in a region of repeats, and 5) occurred at less than 0.01 minor allele frequency (MAF) (in whites) in the Exome Sequencing Project (ESP) database (http://evs.gs.washington.edu/EVS/). Whole genome libraries with ∼280 base inserts were prepared from peripheral blood lymphocyte DNA from Patient 3 according to Illumina'sTruSeq DNA Sample Preparation v2 method (Illumina, San Diego, CA). Libraries were quantitated using KAPA Library Quantification Kit qPCR (KAPA Biosystems, Wilmington, MA), and 500 ng of each of 6 libraries were pooled for capture. The exome capture was preformed according to Illumina's TruSeq Exome Enrichment Kit protocol. Each captured exome pool was sequenced in two lanes on a HiSeq2000 using version 3 chemistry. At least 40 million paired-end 100 base reads were obtained for each sample. Data were processed using RTA ver. 1.12.4.2 and CASAVA 1.8.2. Reads were aligned to a human reference sequence (UCSC assembly hg18, NCBI build 36)using the package called “efficient large-scale alignment of nucleotidedatabases” (ELAND). Reads that aligned uniquely were grouped into genomicsequence intervals of about 100 kb, and reads that failed to align werebinned with their paired-end mates. Reads in each bin were subjected to aSmith-Waterman-based local alignment algorithm, cross_match, using theparameters ­minscore 21 and ­masklevel 0 to their respective 100 kbgenomic sequence (http://www.phrap.org). Genotypes were called at allpositions where there were high-quality sequence bases (Phred-like Q20 or greater) using a Bayesian algorithm (Most Probable Genotype ­ MPG -http://research.nhgri.nih.gov/software/bam2mpg/index.shtml) [52]. In Patients 1 and 2 variants in *LIG4* were verified using Sanger sequencing after PCR amplification of fragments using primers listed in Table S1.

### Determination of LIG4 variant phase

The parents of Patients 1 and 2 were unavailable for this study. To determine if the *LIG4* variants in Patients 1 and 2 were in *cis* or *trans*, 100 ng of peripheral blood lymphocyte DNA was PCR-amplified using the following primers: TTTTGCTCCATGAAACCGAAG (*LIG4* forward) and AGAGTTCAGCACTTGAGCAAAAG (*LIG4* reverse). Greater than 500 ng of each amplimer was sequenced on the PacBio RS instrument (Pacific Biosciences, Menlo Park, CA), following the manufacturer's protocol.

### Determination of copy-number variation

Peripheral blood lymphocyte DNA from Patients 1 and 2 was prepared and hybridized to the Human OmniExpress chip. Genotyping was carried out starting with 200 ng of genomic DNA (4 µl of 50 ng/µl) according to the Illumina Infinium assay protocol [53]. The allele and its intensity were determined using an i-Scan scanner (Illumina), and visualized with the GenomeStudio (Illumina) genotyping module, using human genome build 36 (NCBI36/hg18) for analysis. The genotypes were called using Illumina's cluster file “HumanOmniExpress-12v1_A.egt”. DNA was not available from the parents of Patients 1 and 2. The degree of relatedness for Patients 1 and 2 was determined using a set of population-informative SNPs in a data set that contained 199 samples [54]. Peripheral blood lymphocyte DNA from Patient 3 and his parents was performed using the Illumina Human OmniExpress BeadChip (Illumina). Genotyping was carried out starting with 300 ng of genomic DNA (4 µl of 75 ng/µl) according to the Illumina Infinium assay protocol [53]. The allele and its intensity were determined using a BeadArray scanner (Illumina), and visualized with the GenomeStudio (Illumina) genotyping module, using human genome build 36.1 (NCBI36/hg18) for analysis. The call rates for all the DNA samples were **>**99%. CNVs were detected using cnvPartition 2.4.4 (Illumina) with a confidence threshold of 35 and a minimum probe count of three SNPs.

### Colony survival assay for radiosensitivity

A primary skin fibroblast cell line was established on Patient 1. Whole blood from Patients 2 and 3 was immortalized with Epstein-Barr virus. Lymphoblastoid cells (LCLs) were maintained in RMPI 1640 media and fibroblasts were maintained in DMEM media with 10% fetal bovine serum and 1% penicillin-streptomycin-L-glutamine. LCLs from wild type (normal controls), individuals with ataxia-telangiectasia (A-T) and ligase IV deficiency and Patients 2 and 3 were plated at various concentrations in 96-well plates and subjected to 1 Gy of ionizing radiation; control plates were not irradiated. After two weeks in culture, plates were stained with 0.1% MTT. Wells containing at least one colony of >32 cells were scored as positive. The number of positive wells in irradiated versus non-irradiated plates were compared to determine the survival fraction [13]. Fibroblasts from wild type (normal controls), individuals with A-T, and Patient 1 were plated at various concentrations using 6-well plates and subjected to various doses of ionizing radiation. After two weeks, the plates were stained with 0.1% crystal violet dye and the number of colony forming units was determined for each radiation dose and used to calculate the percent survival fraction relative to the non-irradiated controls.

### Neutral Comet Assay (NCA)

The NCA assay was performed as previously described [14]. Briefly, LCLs from patients, *ATM*-deficient and wild type individuals were exposed to 15 Gy of ionizing radiation using a Mark IV series (Co-137) irradiator at a dose rate of 4.21 Gy/min. Cells were harvested at 30 min and 5 hours post-irradiation. Control cells were not irradiated. Cells were mixed with 1% low-melting-point agarose and plated on a CometSlide (Trevigen, Gaithersburg, MD) then lysed overnight at 4°C. The slides were electrophoresed for 10 min in TBE buffer at 21 V. They were then washed with water and 70% ethanol. After drying, the slides were stained with 0.1% SYBR Gold (Invitrogen, Carlsbad, CA) and visualized using a Zeiss fluorescent microscope equipped with an AxioVision camera. Comet Score software (TriTek Corp, Sumerduck, VA) was used to calculate the tail moment for each cell. The average tail moment of >50 cells was calculated for each condition. The experiment was performed in triplicate.

### Western blotting of ligase IV

Western blots were loaded with 50 micrograms of whole-cell lysate (in radio-immunoprecipitation assay buffer) from LCLs or fibroblasts, along with normal and ligase IV-deficient controls. The gels were developed with commercial anti-ligase IV antibody (Abcam ab80514, 1∶1000, Abcam, Cambridge, MA, USA). Antibody to GAPDH (1∶3000, Novus Biologicals, Littleton, CO, USA) was used as a loading control [55].

The content of this publication does not necessarily reflect the views or policies of the Department of Health and Human Services, nor does mention of trade names, commercial products or organizations imply endorsement by the U.S. Government.

## Supporting Information

Figure S1 **Cryptic relatedness analysis.**
(DOCX)

Table S1 **List of primers for Sanger sequencing of** ***LIG4*** **.**
(XLSX)

Table S2 **Determination of** ***cis*** **and** ***trans*** **status of** ***LIG4*** **variants in Patients 1 and 2.**
(XLSX)

Table S3 **Variants identified by exome sequencing of Patient 3 (and parents).**
(XLSX)

Table S4 **Variants in deletion of chromosome 17q24.3 in Patient 3, as identified by exome sequencing.**
(XLSX)

Table S5 **Literature review of patients with** ***LIG4*** **mutations.**
(XLSX)
