# Supplementary material for: Dubowitz Syndrome Is a Complex Comprised of Multiple, Genetically Distinct and Phenotypically Overlapping Disorders
Source: PLoS One. 2014 Jun 3;9(6):e98686. doi: 10.1371/journal.pone.0098686 (PMC4043752; doi:10.1371/journal.pone.0098686)
Supplement: Figure S1 — Cryptic relatedness analysis. (DOCX) [file pone.0098686.s001.docx]

# Supporting Figure 1

# Data Processing

Patients 1 and 2 were genotyped on the Illumina HumanOmniExpress-12v1_A chip from two different scans. Genotypes were called using Illumina’s cluster file “HumanOmniExpress-12v1_A.egt”. Data was exported from Illumina’s GenomeStudio software using their “Genotype Final Report” (GFR) format. Using the GFR file as input, a high-performance binary file format (GDAT) was generated.

The completion rate was then estimated using GLU. Table 1 is the completion rate by sample.

# Table 1 Completion rate by sample

| ASSAY_ID | Completion Rate (%) |
| --- | --- |
| Patient 1 | 98.99 |
| Patient 2 | 99.12 |

# Relatedness Check

The close relationships (1-2^nd^ degree) were computed using a set of population informative SNPs (Kai Yu et al. PLoS ONE 2008) in a data set containing 199 samples. The PIHAT = 0.5631 between these two samples is consistent with a full-sibling relationship between them. Table 2 summarized the results.

**Table 2 Cryptic relatedness**

| SAMPLE1 | SAMPLE2 | COMPARISONS | IBD0 | IBD1 | IBD2 | PIHAT |
| --- | --- | --- | --- | --- | --- | --- |
| Patient 2 | Patient 1 | 8011 | 0.2196 | 0.4346 | 0.3458 | 0.5631 |

These results are consistent with the 2 samples being full-sibs.

# Autozygosity Analysis

The Intensity plots were generated for each chromosome on gdat file. No obvious segments of autozygosity were detected for either of the samples. These observations indicate that these 2 individuals are not products of a consanguineous mating. The plot for the X chromosome shows Patient 1 is male. Patient 2 is female. See Figure 1a and 1b.


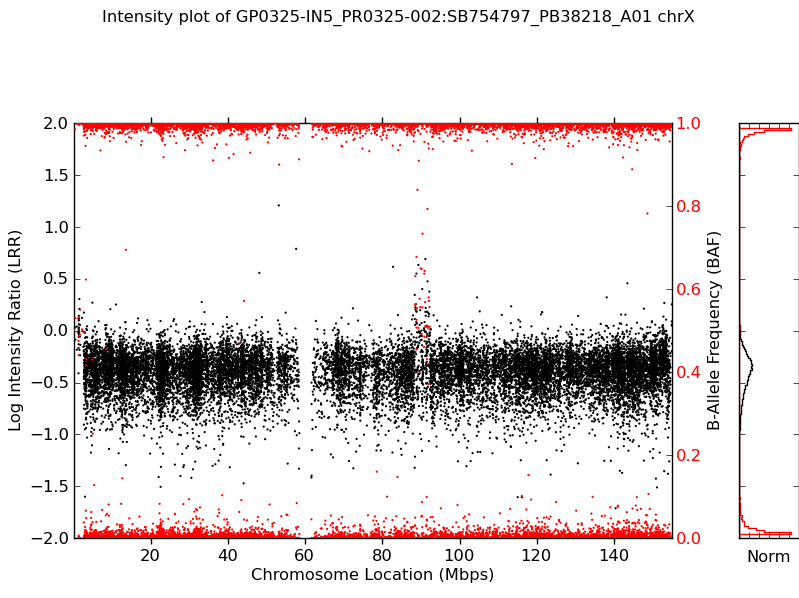


Figure 1a: Male gender characterized by a black hemizygous AB allele band on chromosome X.


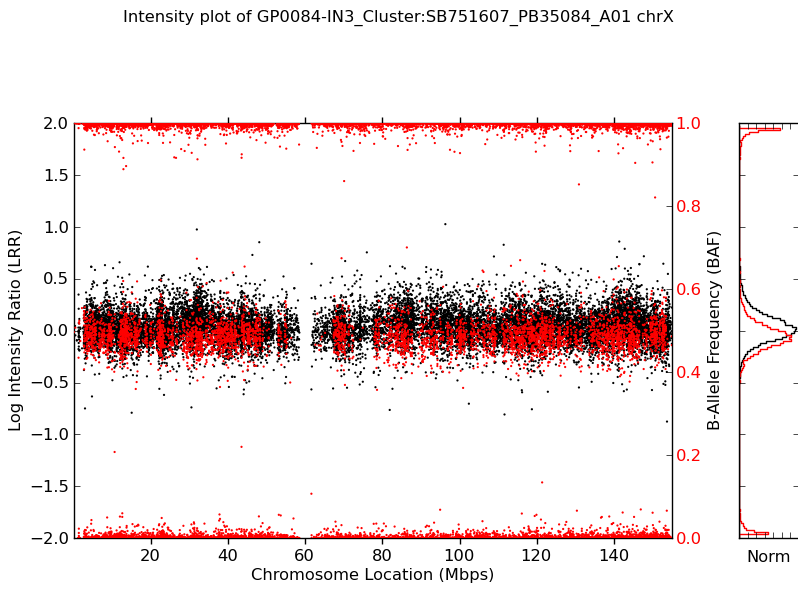


Figure 1b: Female gender characterized by red and black heterozygous AB allele bands on chromosome X.
